# Supplementary material for: MoDLE: high-performance stochastic modeling of DNA loop extrusion interactions
Source: Genome Biol. 2022 Nov 30;23:247. doi: 10.1186/s13059-022-02815-7 (PMC9710047; doi:10.1186/s13059-022-02815-7)
Supplement: Supplementary file 2 — Additional file 2. Supplementary figures. Supplementary Figures 1-12. [file 13059_2022_2815_MOESM2_ESM.pdf]

MoDLE: High-performance stochastic modeling of DNA loop extrusion interactions

Supplementary figures

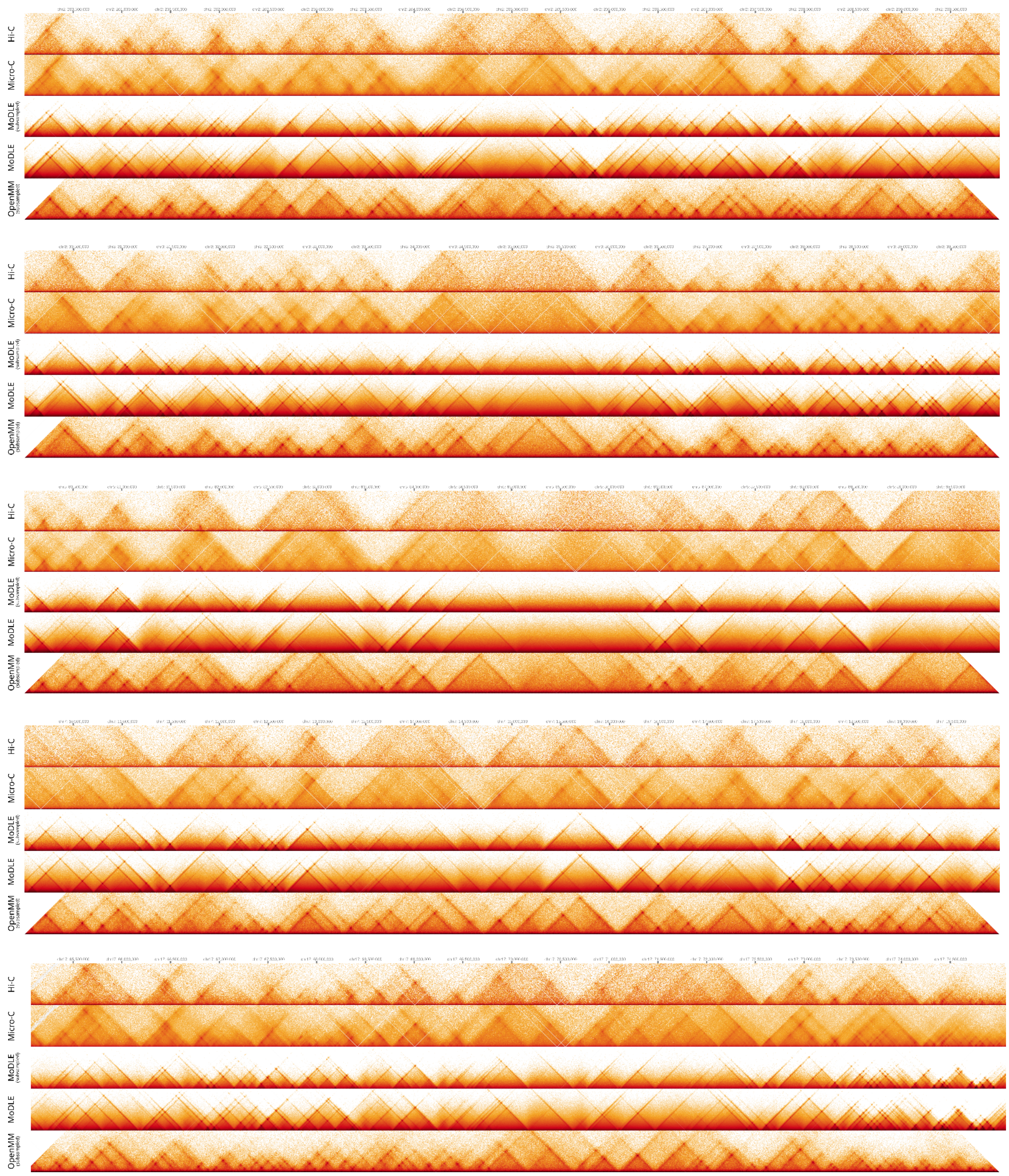

Fig. S1: Side-by-side comparison of Hi-C and Micro-C data (top two panels), MoDLE output with

and without subsampling (third and fourth panels respectively) and OpenMM output (bottom panels for all selected 10Mbp simulation regions on chromosomes 2, 3, 5, 7 and 17).

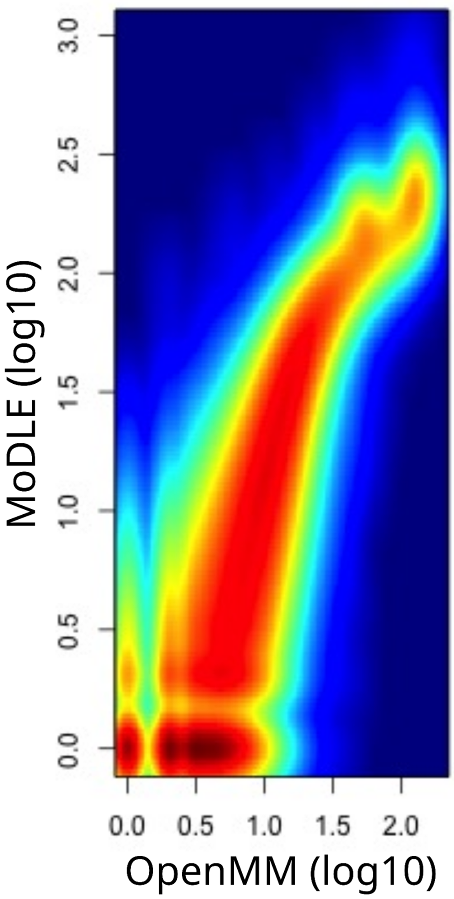

Fig. S2: Density plot showing frequency (blue = low; red = high) of pixel values in MoDLE (horizontal axis; log10-transformed) vs. OpenMM (vertical axis; log10-transformed). Pearson correlation coefficient is ρ=0.93.

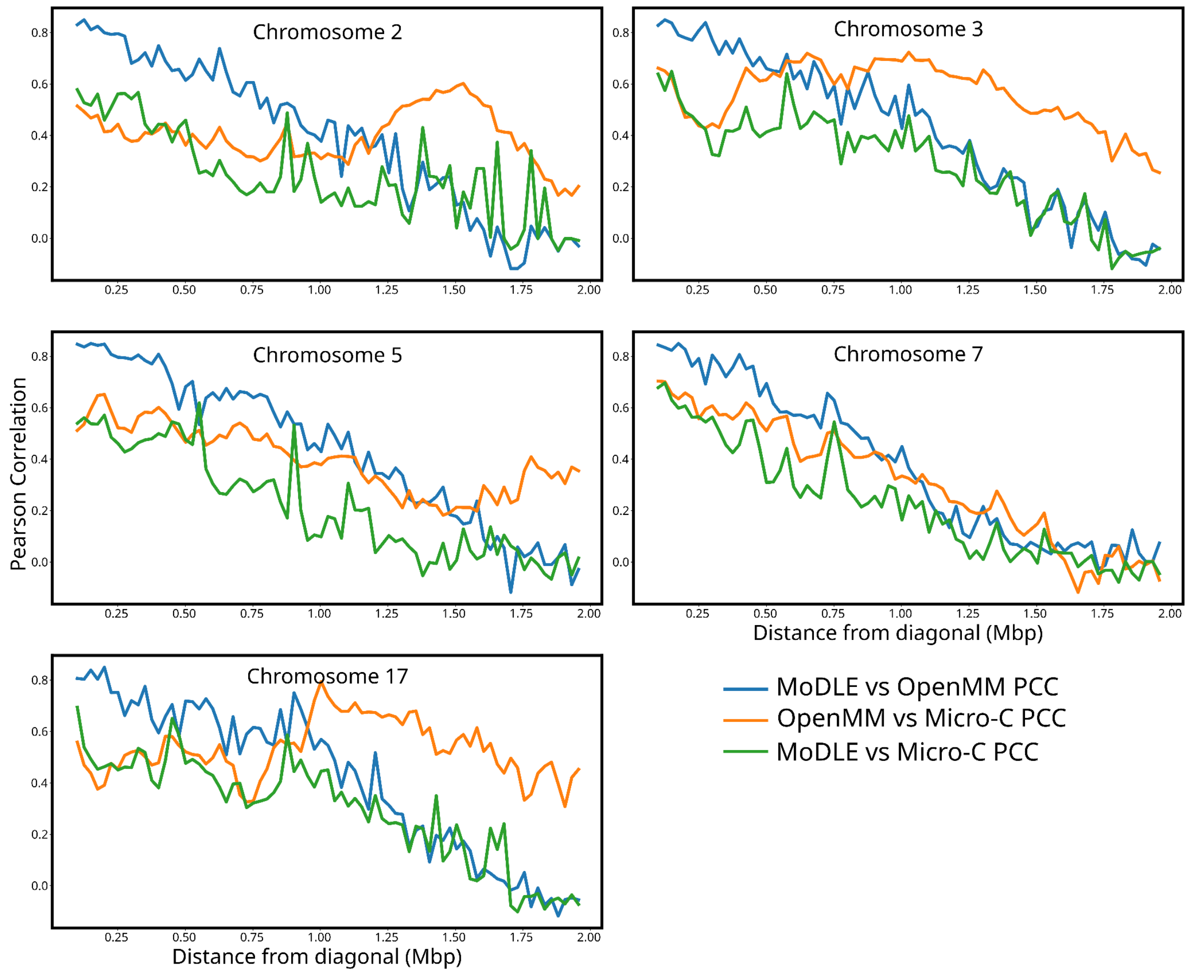

Fig. S3: Diagonal-by-diagonal correlation plots. Each panel shows the Pearson correlation of MoDLE vs. OpenMM (blue), OpenMM vs. Micro-C (yellow), and MoDLE vs. Micro-C (green) for all selected 10Mbp simulation regions on chromosomes 2, 3, 5, 7 and 17). The correlation is shown for all contact map sub-diagonals from the diagonal to 2Mbp from the diagonal (horizontal axes).

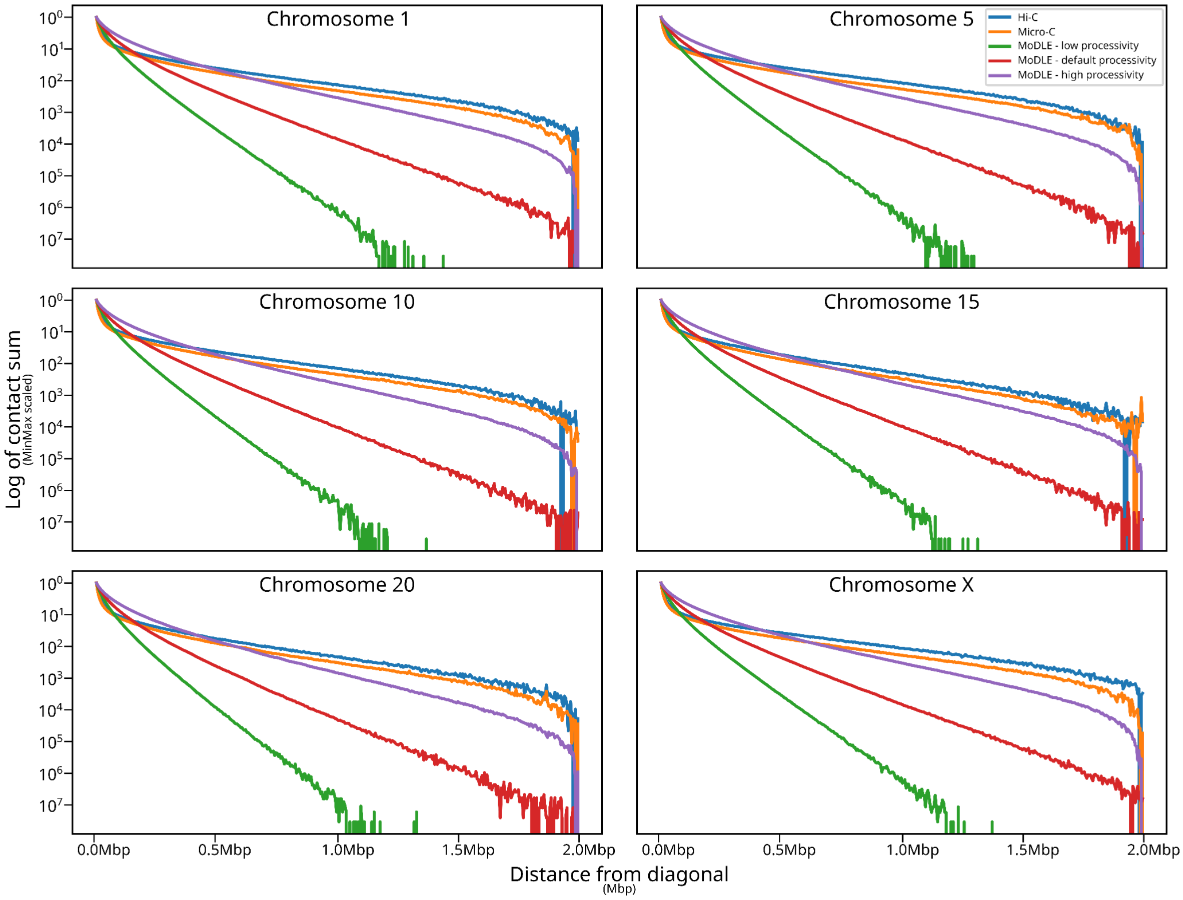

Fig. S4: Relationship between distance from diagonal (Mbp) and contact frequencies (log of contact sum) for hESC Hi-C data (blue), hESC Micro-C data (yellow), MoDLE using low processivity (150 kbp; green), MoDLE using default processivity (300 kbp; red), and MoDLE using high processivity (600 kbp; purple).

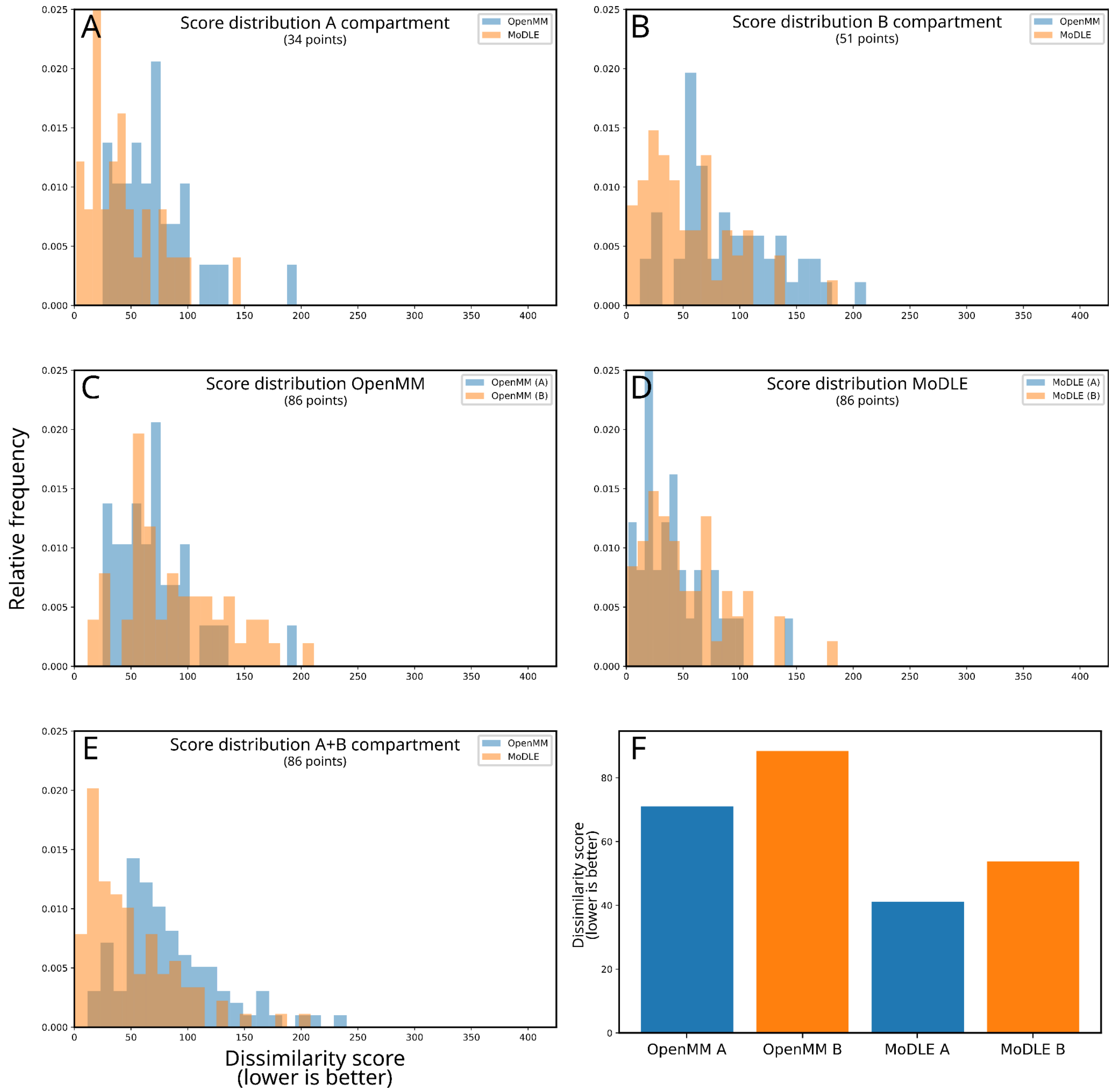

Fig. S5: Comparison of mean and distribution of dissimilarity scores of MoDLE and OpenMM simulations using H1 hESC Micro-C as reference.
Panel A and B contrast score distribution for MoDLE and OpenMM simulated matrices in A and B compartments respectively.
Panel C and D contrast score distribution of A and B compartments for OpenMM and MoDLE simulated matrices respectively.
Panel E shows the score distribution for OpenMM and MoDLE simulated matrices, regardless of compartment.
Panel F shows the average score for OpenMM and MoDLE simulations in A and B compartments.
Scores are computed as described in Methods (part 6). Before intersecting scores with compartment information, scores are filtered by taking the intersection of the score themselves, the architectural stripes on the reference matrix (stripes annotated with Stripenn) and the genomic regions simulated with OpenMM (chr2:200-210Mbp, chr3:30-40Mbp, chr5:80-90Mbp, chr7:10-20Mbp and chr17:65-75Mbp).

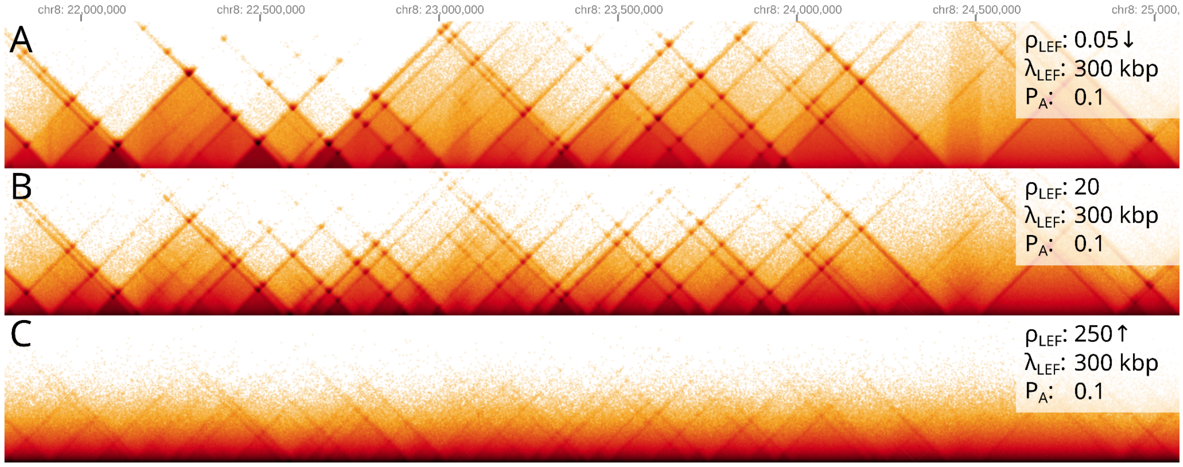

Fig. S6: Simulated heat maps showing the effect of increasing LEF density (ρ_{LEF}). Higher LEF densities cause contacts to distribute closer to the diagonal. This is due to an increased probability of LEF-LEF collisions. Furthermore, at very high ρ_{LEF} (panel C), stripes and dots appear less defined. This can once again be explained by an increased rate of LEF-LEF collisions, which are stochastic in nature, and do not necessarily occur at or near extrusion barriers.

Legend:
ρ_{LEF}: LEF density expressed as the number of LEFs per Mbp of simulated DNA
λ_{LEF}: Average LEF processivity
P_A: Probability of LEF-LEF collision avoidance

↓: Parameter is smaller than the default value
↑: Parameter is larger than the default value

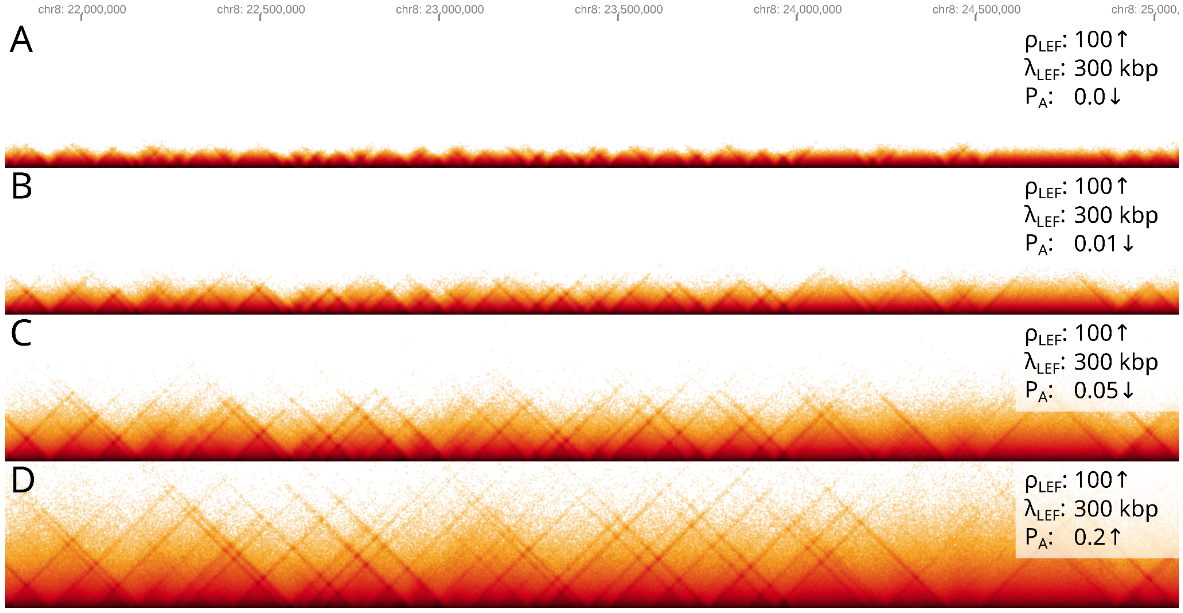

Fig. S7: Simulated heat maps showing the interplay between LEF density (ρ_{LEF}) and probability of LEF-LEF collision avoidance (P_A).
This scenario further expands what is shown in Fig. S4, and shows how increasing P_A can partially counteract very high LEF densities.

Legend:
ρ_{LEF}: LEF density expressed as the number of LEFs per Mbp of simulated DNA
λ_{LEF}: Average LEF processivity
P_A: Probability of LEF-LEF collision avoidance

↓: Parameter is smaller than the default value
↑: Parameter is larger than the default value

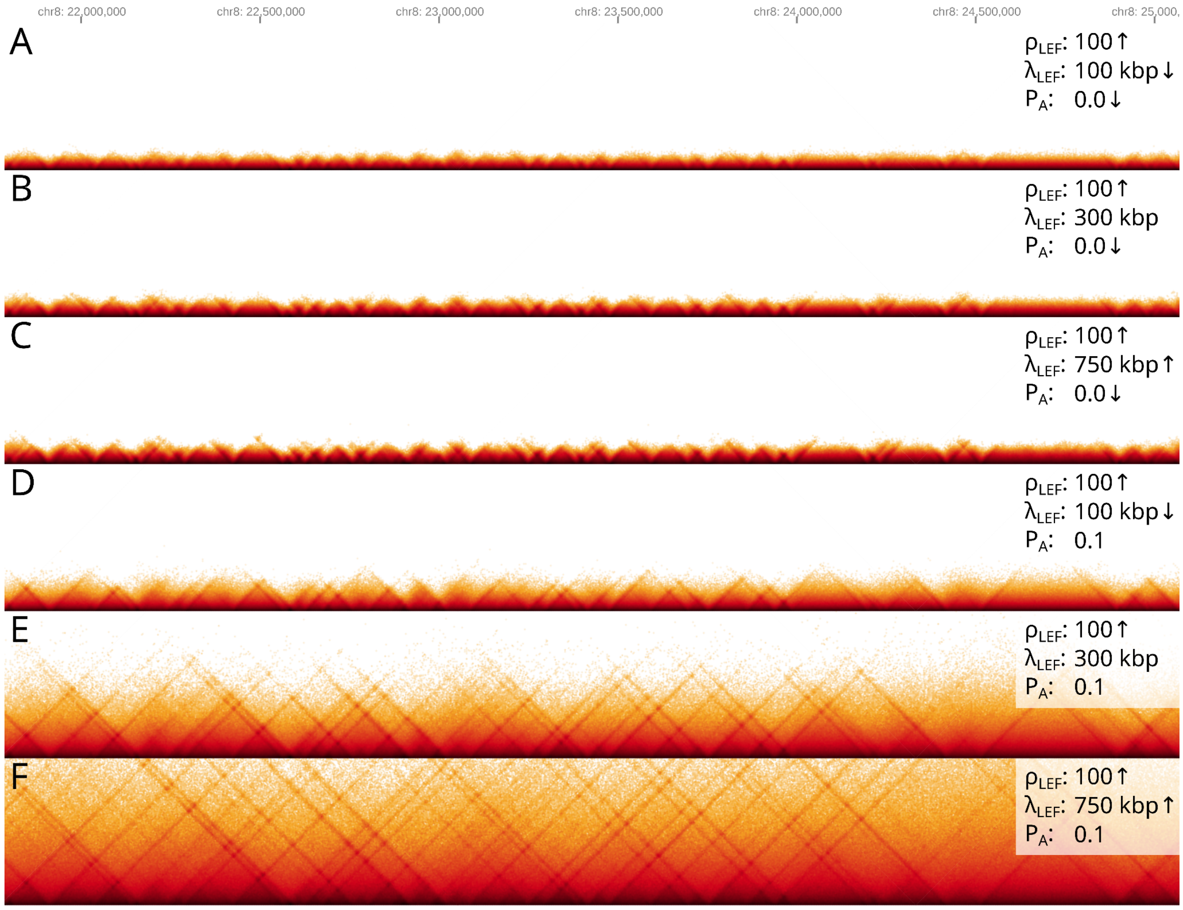

Fig. S8: Simulated heat maps showing the effect of (dis)allowing LEF-LEF collision avoidance at increasing levels of LEF processivity (λ_{LEF}).
Increasing λ_{LEF} has virtually no effect when the rate of LEF-LEF collisions is high and collisions are deterministic (i.e. LEF-LEF collisions cannot be avoided). Allowing LEFs to bypass one another with a relatively low probability is sufficient to observe progressively longer loops produced by increasingly higher λ_{LEF}.

Legend:
ρ_{LEF}: LEF density expressed as the number of LEFs per Mbp of simulated DNA
λ_{LEF}: Average LEF processivity
P_A: Probability of LEF-LEF collision avoidance

↓: Parameter is smaller than the default value
↑: Parameter is larger than the default value

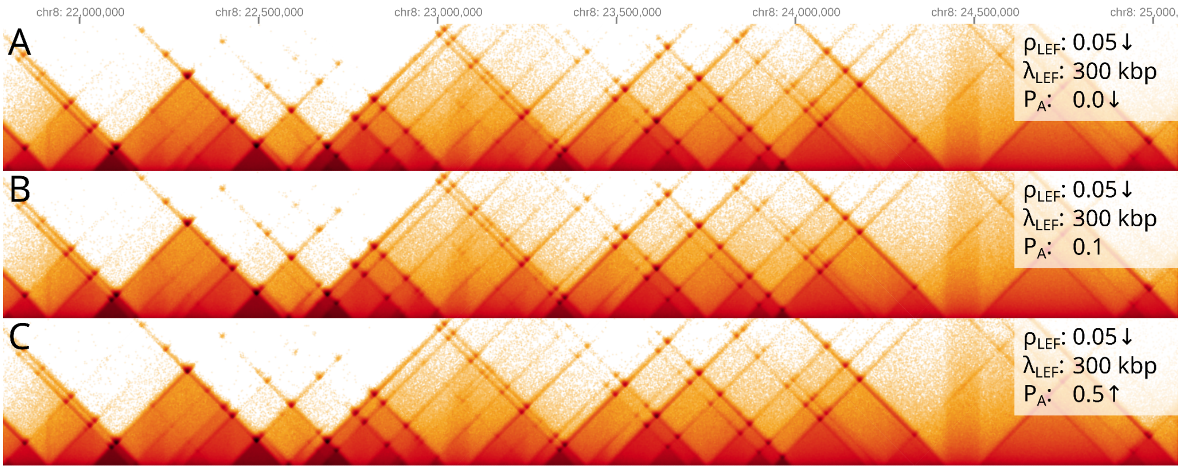

Fig. S9: Simulated heat maps showing the consequences of simulating with very low LEF densities (ρ_{LEF}).

When simulating with very low ρ_{LEF}, the probability of two or more LEFs extruding the same region at the same time is approximately 0. Thus, increasing P_A has no effect in practice.

This scenario also highlights one of the consequences of MoDLE simulations not accounting for polymer physics. Very low ρ_{LEF} would likely result in no appreciable contact enrichment due to loop extrusion in vivo or in MD polymer simulations, as once a loop has been extruded, there is plenty of time for the chromatin polymer to relax before the another LEF extrudes through the same region. This is not the case in MoDLE, as loops cease to exist as soon as LEFs are released.

Legend:

ρ_{LEF}: LEF density expressed as the number of LEFs per Mbp of simulated DNA

λ_{LEF}: Average LEF processivity

P_A: Probability of LEF-LEF collision avoidance

↓: Parameter is smaller than the default value

↑: Parameter is larger than the default value

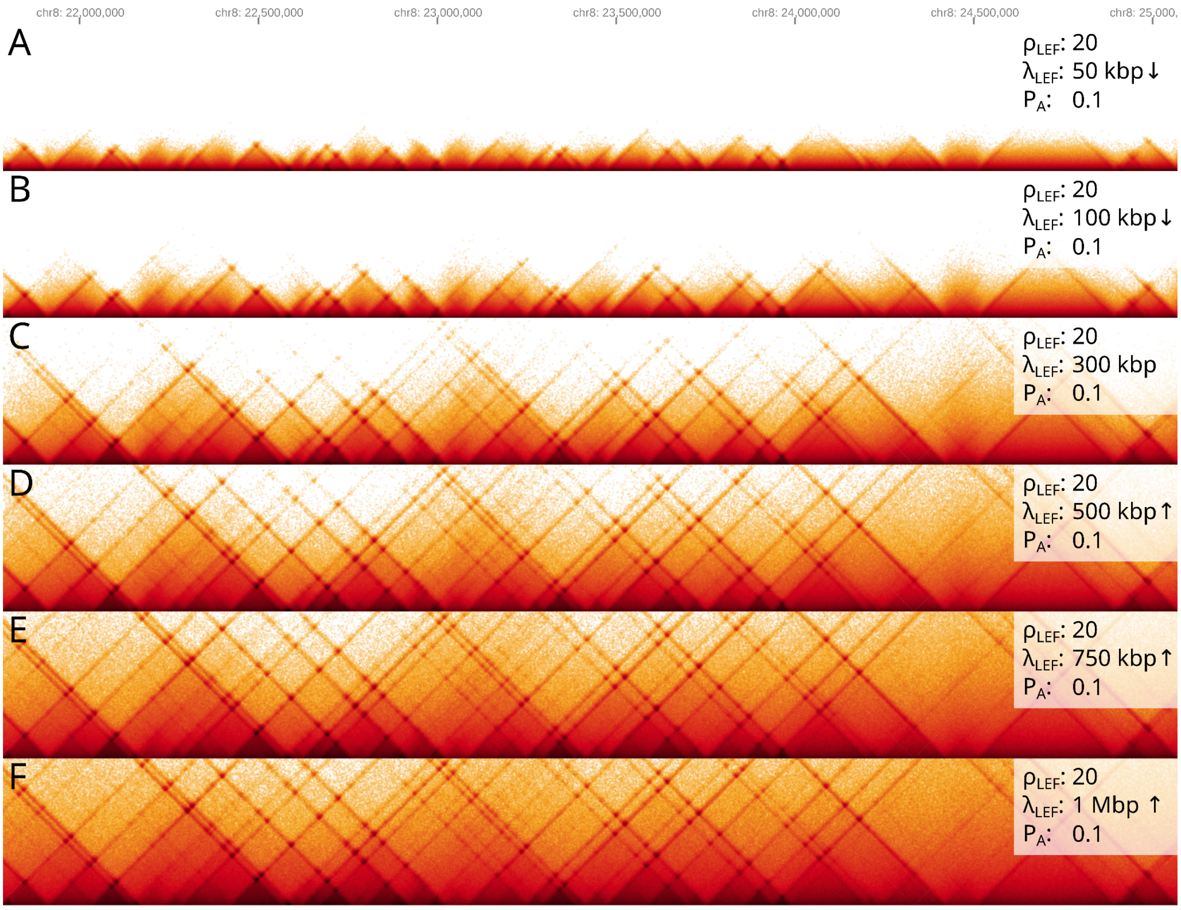

Fig. S10: Simulated heat maps showing the effect of different LEF processivities (λ_{LEF}) when LEF density (ρ_{LEF}) and probability of LEF-LEF collision avoidance (P_A) are left at default.
With realistic LEF concentrations, altering λ_{LEF} has the expected effect: higher processivities lead to loops, which result in longer stripes and stronger dots even at relatively high distances from the diagonal.

Legend:
ρ_{LEF}: LEF density expressed as the number of LEFs per Mbp of simulated DNA
λ_{LEF}: Average LEF processivity
P_A: Probability of LEF-LEF collision avoidance

↓: Parameter is smaller than the default value
↑: Parameter is larger than the default value

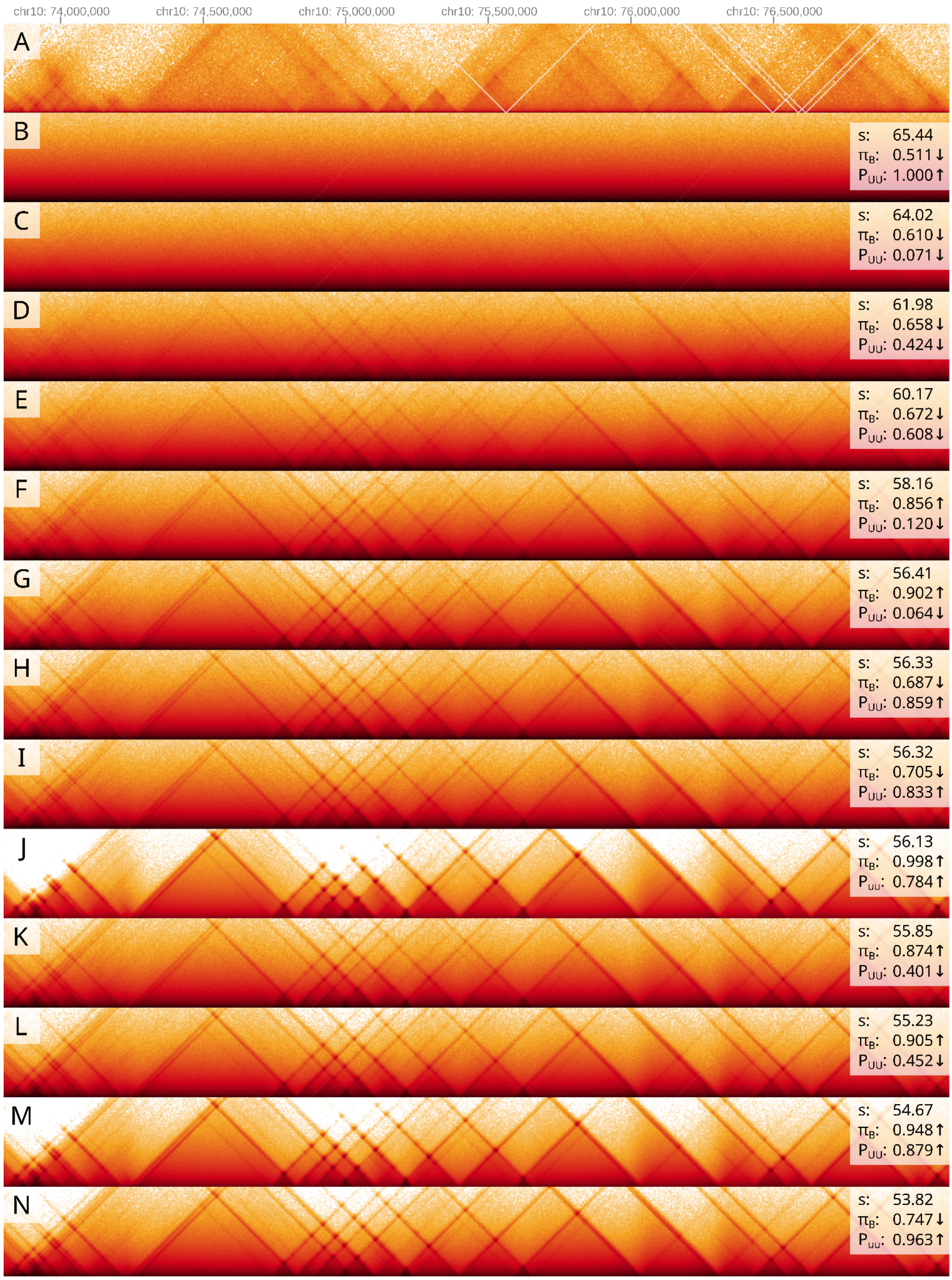

Fig. S11: Side-by-side comparison of H1-hESC Micro-C data (top panel) and matrices simulated by MoDLE. Panels B-N show a selection of the parameter combinations explored by the Gaussian optimization described in Methods (part 9).
On the right, each panel is labelled with its corresponding score s, occupancy π_B and self-transition probability of the unbound state P_{UU}.

Panels are sorted by score in descending order (lower scores are better).
Upwards and downwards arrows next to parameter values indicate whether a value is higher or lower than the default parameter.

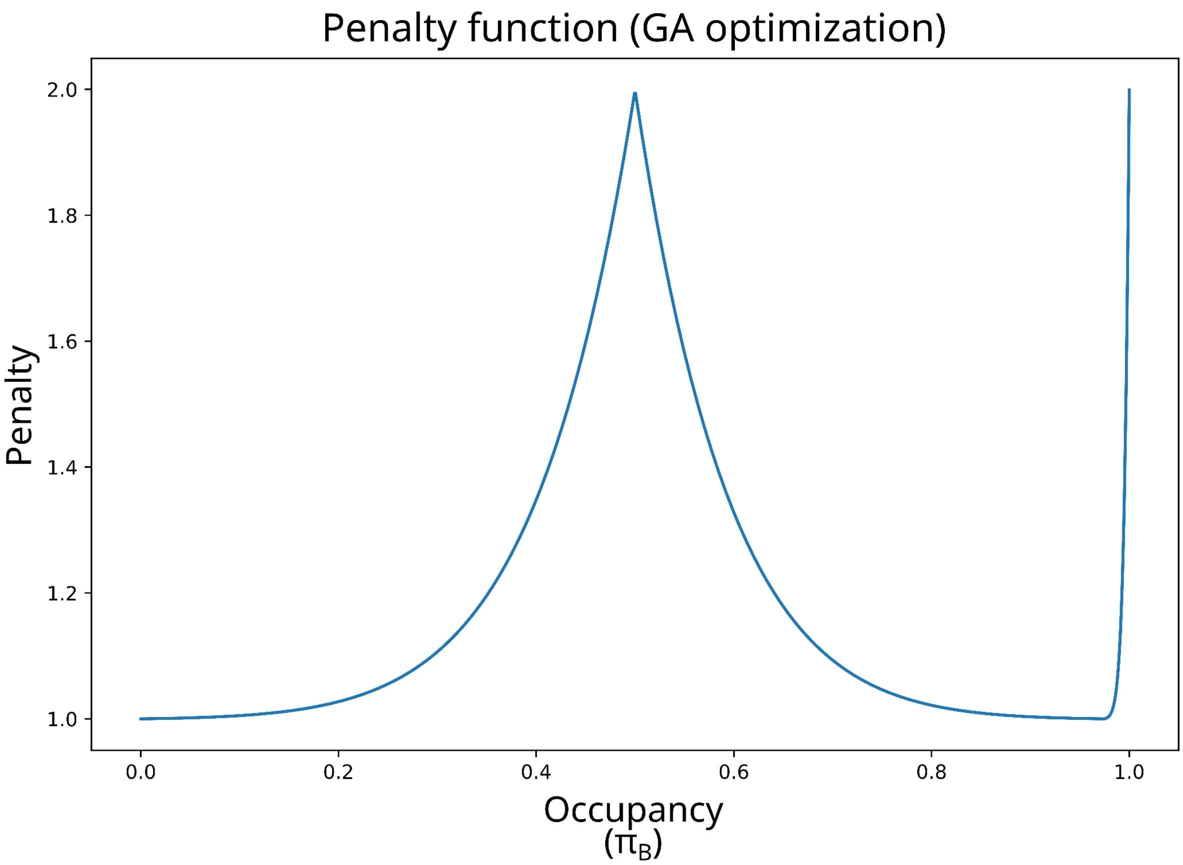

Fig. S12: Plot of the penalty function used in the GA optimization of extrusion barrier parameters.
