# Supplementary material for: MoDLE: high-performance stochastic modeling of DNA loop extrusion interactions
Source: Genome Biol. 2022 Nov 30;23:247. doi: 10.1186/s13059-022-02815-7 (PMC9710047; doi:10.1186/s13059-022-02815-7)
Supplement: Supplementary file 3 — Additional file 3. Supplementary tables. Supplementary Tables 1-2 [90–93]. [file 13059_2022_2815_MOESM3_ESM.pdf]

MoDLE: High-performance stochastic modeling of DNA loop extrusion interactions

Supplementary tables

Table S1: Parameter table for MoDLE simulations used to generate Fig. 2.

Figure	Bin size (bp)	Extrusion barrier occupancy	Target contact density	# of LEFs per Mbp	Diagonal width (Mbp)	Avg. LEF processivity (kbp)	Contact sampling strategy
2A	5000 (default)	Variable	20.0	20 (default)	3 (default)	300 (default)	Loop only with noise
2B	5000 (default)	Variable	20.0	20 (default)	3 (default)	300 (default)	TAD only with noise
2C, 2D, 2F (WT)	5000 (default)	Variable	20.0	20 (default)	3 (default)	300 (default)	TAD plus loop with noise (default)
2F ΔCTCF	5000 (default)	0.2	20.0	20 (default)	3 (default)	300 (default)	TAD plus loop with noise (default)
2F ΔWAPL	5000 (default)	Variable	10.0	20 (default)	6	3000	TAD plus loop with noise (default)

Table S2: List of MoDLE software dependencies.

Name	Project URL	Version	License
Abseil C++	abseil.io	20211102 LTS	Apache 2.0
bitflags	github.com/m-peko/bitflags	1.5.0	MIT
Boost C++	boost.org	1.79.0	BSL 1.0
bzip2	sourceware.org/bzip	1.0.8	BSD-like

	2		
Catch2	github.com/catchorg/Catch2	3.1.0	BSL 1.0
CLI11	github.com/CLIUtils/CLI11	2.2.0	BSD 3
cmake-git-version-tracking	github.com/andrew-hardin/cmake-git-version-tracking	N/A	MIT
concurrentqueue	github.com/cameron314/concurrentqueue	1.0.3	Simplified BSD
cpp-sort	github.com/Morwenn/cpp-sort	1.13.0	MIT
fast_float	github.com/fastfloat/fast_float	3.5.1	MIT
fmt	github.com/fmtlib/fmt	9.0.0	MIT
HDF5 [90]	hdfgroup.org	1.12.2	BSD 3
libarchive	libarchive.org	3.6.1	BSD 2
libBigWig	github.com/dpryan79/libBigWig	0.4.7	MIT
libcuckoo [91,92]	github.com/efficient/libcuckoo	0.3.1	Apache 2.0
LZ4	lz4.org	1.9.3	BSD 2
LZO	oberhumer.com/opensource/lzo	2.1.0	GPL 2.0
range-v3	github.com/ericniebler/range-v3	0.12.0	BSL 1.0
readerwriterqueue	github.com/cameron314/readerwriterqueue	1.0.6	Simplified BSD
spdlog	github.com/gabime/spdlog	1.9.2	MIT
bshoshany-thread-pool [93]	github.com/bshoshany/thread-pool	3.3.0	MIT
TOML++	marzer.github.io/tomlplusplus	3.1.0	MIT
Xoshiro-cpp	github.com/Reputele	1.1	MIT

	ss/Xoshiro-cpp		
xxHash	xxhash.com	0.8.1	BSD 2
XZ Utils	tukaani.org/xz	5.2.5	GPL 3.0
zlib	zlib.net	1.2.11	Zlib
zstd	github.com/facebook/zstd	1.5.2	BSD 3
