# Supplementary material for: MoDLE: high-performance stochastic modeling of DNA loop extrusion interactions
Source: Genome Biol. 2022 Nov 30;23:247. doi: 10.1186/s13059-022-02815-7 (PMC9710047; doi:10.1186/s13059-022-02815-7)
Supplement: Supplementary file 4 — Additional file 4. Review history. [file 13059_2022_2815_MOESM4_ESM.docx]

**1st round**

**Reviewer 1**

Transparency statement: this review was written by Guillaume Filion (University of Toronto Scarborough). I accepted the assignment on June 14 2022 and mentioned to the Editor that I would probably not be able to turn in my review within 10 days because I was quite busy. Unrelated to this, on June 27 2022 I contacted the Editor to request extra time because of an electrical accident at the university that prevented me from using my computer for several days. The incident was fixed on July 6 2022; I finished running the tests on July 7 2022 and submitted my review on the same day. I have worked approximately 7 hours on this review. I reviewed the text and I tested the software tool but I did not inspect the code. I declare no conflict of interest and I feel qualified to review the manuscript.

In the article titled "MoDLE: High-performance stochastic modeling of DNA loop extrusion interactions", Rossini et al. propose a software tool to simulate Hi-C maps generated by the process of loop extrusion.

MoDLE simulates a Markov chain whereby loop-extrusion factors and barriers are assumed to bind / unbind idealized chromosomes and to extrude it at every iteration, while detecting collisions and resolving them. MoDLE is implemented in C++ and is optimized for speed and resource frugality. MoDLE is at least an order of magnitude faster than simulations based on molecular dynamics. The main reason is that the mechanics are simpler and therefore less costly to simulate, but the authors also optimized the code and the data structures with concepts such as cache friendliness, data contiguity etc.

The authors benchmark MoDLE for accuracy, speed and scalability against state-of-the-art OpenMM tools with favorable results for MoDLE. Finally, the authors demonstrate how to use MoDLE for inference when CTCF ChIP-seq profiles are not available.

I have learned a lot while reading this manuscript. I had some notions of the methods that are used to simulate chromosome dynamics (PMID:29712907) but I was not aware of many technicalities. Also, I was not aware of the concept of Bayesian optimization, which I found very interesting.

Overall, the quality of the work is very high. I did not find a single grammatical error. The manuscript is very clear, the implementation of the software is solid, the manual pages are clear and the presented information is relevant. I only have comments about one section.

I recommend the authors to address the comments below in order to improve the manuscript.

SCIENCE

1. There is no real biological insight in the manuscript. The Authors do not really explain how MoDLE could be useful to understand the underlying biological processes. They advertise their tool as a fast alternative to existing tools in the field; they show how it can be used to make predictions but there is no strong message about inference. It is up to the Editor whether some biology is required. My opinion is that the article would be better if the Authors explained what question could be answered with MoDLE, and greatly better if they answered some of those questions.

2. It seems to me that the Authors could slightly modify their work in section "Genome wide parameter optimization" to somehow address the point above. If they let every CTCF site have their own value of P_BU (the dissociation rate), and fitted those values using the same Bayesian optimization procedure, they would obtain a CTCF binding profile. This is something they could compare to the actual profile and thereby verify that their model does allow to infer the underlying biology. This is not the best application because CTCF ChIP-seq is easy and Hi-C is hard, but at least this would show whether MoDLE can be used for inference in biology.

TEXT

The section "Genome wide parameter optimization" generated a lot of confusion in my mind.

1. Line 243: "Notably, the results show that the near-optimal CTCF binding probabilities have values typically >0.8, except when the stability of binding (P) is high…"

What does that mean? The optimal value is 0.694, so what is typical about values greater than 0.8. Do the authors simply mean that when pi_B increases P_UU decreases? If so, why the complicated logic flow?

2. Line 245: "... supporting a dynamic exchange model of CTCF binding and a transient maintenance of loops [42]".

How is that supporting this model exactly? The optimal value of P_UU 0.978 is close to the maximum possible so how is this dynamic? In other words, what kind of outcome would have been against a dynamic exchange model?

3. Line 246: "Thus, CTCFs can potentially compensate for a lower CTCF binding probability by increasing "traffic jams" between extruding LEFs".

How is this a consequence of the previous statement? Also, I have a hard time understanding what the sentence means, starting from CTCF compensating for its binding probability, to the comparison lower with respect to what and finally the use of traffic jams between quotes, which seems to refer to a situation that is loosely defined.

Figure 4A adds to the confusion. The two series of squares seem to represent the binding state of CTCF at barriers. Why are the bound sites more aggregated when P_UU + P_BB are high? I understand that the system becomes more static (fewer CTCF changes per epoch) in this case but according to the description of the model, CTCF binding is space-less (section 6 of supplementary document 1). So I do not understand how the spatial aggregation of occupancy comes about. I understand that when P_UU + P_BB are high, loop-extrusion factors spend more time at the barriers and may aggregate there, but this is not what the profiles are meant to represent (the legend says that it is the stationary distribution of pi_B).

FIGURES

1. Figure 2E: The authors should describe what "Accuracy" is.

2. Figure 4B: The authors should say something about the y axis. I understand that this is the standard output of scilearn-kit and the score function is fairly complex, but the plot is meaningless without this information. At the very least, the authors should link to the section describing the objective function.

CODE

I ran into some issues trying to compile and install the code. I have raised them on the Github page of the project. I mention them here briefly for the record.

1. The Conan troubleshooting info should be much more visible.

2. The dependence on h5diff to run the test is not explicit in the manual.

IRRELEVANT STUFF

The comments below do not require a response; they are just the expression of personal opinions that are not relevant for the review but may still be useful to whomever reads them.

1. I wasted quite a lot of time because at the time I downloaded the code, the last commit on the main branch was 423e279. Compilation worked, but simulations did not return even after several hours using multiple fast CPUs. When I resumed work, I realized that there were 63 commits since I downloaded the code on June 14. I checked out the commit of v1.0.0-rc.6, recompiled everything, which solved the issue. It is better practice to push non-release commits to separate branches and merge them with main only when ready. When submitting a code for review, it would also clarify which version of the code has to be reviewed and which are future developments.

2. I really appreciate that the figures are at the right place in the text and not at the end. The practice of separating the figures from the text and the legend from the figures costs a surprising amount of Reviewer time and I think the Authors for arranging the document the way they did. I hope that they will maintain this habit.

**Reviewer 2**

In their work, the authors describe MoLDE, a computational method to model DNA contacts based on the loop extrusion model. According to the authors, the method is able to efficiently simulate contact maps of extended regions (several Mb genomic distance spanned) based on input files (i.e. CTCF and RAD21 data) containing biological information necessary to perform the simulation or by optimizing the model parameters from MicroC data.

In general, this is an important topic in the context of a constantly expanding research field. Therefore, the work is timely and could be useful for the community. Also, the algorithm is fully provided and useful to model biologically relevant contacts event driven by CTCF and Cohesin and to dissect the higher-order organization of chromatin. I found the work interesting and I appreciate the methodology that finds the LE contact maps through an optimization algorithm, providing an original perspective for an interesting question of chromatin architecture field. Nevertheless, several points discussed in the paper need to be better considered and expanded.

An important concern is about the scientific novelty of the results obtained by the presented MoLDE and I do not see any improvement of the state-of-the-art as the LE model in itself and its variants is widely studied in literature. In this regard, I would suggest to expand the scientific applications of the tool and provide more evidence to support its versatility. So far, I see just figure 2 dedicated to some very basic applications. A specific list o point follows:

1) It would be interesting to present an application to evaluate the importance of different molecular mechanisms in relevant genomic loci. Specifically, it would be worth to consider other aspects known to be fundamental to chromatin architecture: what about phase-separation? Does the lack of this mechanism affect the accuracy of the prediction? Is the tool able to tell something about the role of each mechanism (LE and phase-separation) in some specific, biologically relevant examples?

2) How the MoDLE predictions are related to other chromatin features, as A/B compartments?

3) What about gene-enhancer contacts? Are there loci where the model can tell something about specific regulatory elements?

4) Does the model correctly recapitulates contact probability decay? Is there a critical genomic distance above which predictions are less reliable?

5) I would evaluate the accuracy of the algorithm with other tests, e.g. if mutants phenotypes at a particular genomic distance can be correctly predicted.

6) Technical observations are about the evaluation of the accuracy. I think that other metrics should be used (distance corrected correlations, HiCRep from Yang et al 2017, correlations curve diagonal by diagonal) to estimate the similarity between MoDLE and experiments.

7) Also, more investigations about MoDLE output in varying the main LE parameters, i.e. LE density and processivity: how is the change of the pattern?

8) Is the tool able to find the best LE parameter arrangement for a specific locus? Does the model result match with biological estimates of such quantities about Cohesin density and LEf processivity?

9) Supp. 1 seems that long range is not well captured? Please comment on that.

10) Beyond MicroC, a comparison with other data, such as HiC, would be useful?

11) Add some synthetic and specific info in the page 7. How is the combination TAD contact - LE done? Please add some more details here with reference to the Methods.

**Reviewer 3**

Please consider the following questions:

-Is the Software likely to be of broad utility? Is it easy to install and use?

Maybe

-Does the software represent a significant advance over previously published tools (as demonstrated by direct comparison with available related software)?

No

-Please indicate briefly the novel features and/or advantages of the software and/or please reference the relevant publications and which alternatives, if any, it should be compared with.

see below

-Are the methods appropriate to the aims of the study, are they well described, and are necessary controls included?

No.

-Are the conclusions adequately supported by the data shown?

Yes, but there are little conclusions.

-Are sufficient details provided to allow replication and comparison with related analyses that may have been performed?

yes

-Is the paper of broad interest to others in the field, or of outstanding interest to a broad audience of biologists?

No

The authors introduce a high-performance stochastic model called Modeling of DNA Loop Extrusion (MoDLE).

Building on OpenMM, they developed an efficent model to simulate loop extrusion dynamics and evaluate contacts matrix along genome.

The paper is particularly focus on the performance of this model and on thecnical details to prove that MoDLE is a more suitable choise compared to OpenMM. Only in the session "Comparison with Micro-C data and MD simulations" (and fig.2), the authors present the results of their model on five different 10 Mbp regions on five different chromosomes where the comparison with biological data seems limited to the median pixel accuracy.

The part of the model involving loop extrusion and CTCF introduces parameters for self-transitions and probability of a given CTCF to be occupied. The model can infer these parameters from ChIP-seq data or evaluate them using a Bayesian optimization. These parameters are not well contestualized in the biological dataset.

It is not clear what are the real differences between MoDLE and OpenMM: the authors present a comparison limited to the computational performances. What are the differences in terms of the model? Are their model for loop extrusion compatible with the one in OpenMM? What about the dynamical properties?

This paper is really thecnical, focus in the perfomance improvements with a brief comparison with the biological context, It is not approrpaite for genome biology and should be send to focus journal dealing with software comparison performance. It is not for a broad audience of biologists.

**Authors’ response**

Reviewer #1

We thank the reviewer for the positive and constructive feedback and for the suggestions for improving the manuscript. See below for our point-by-point response to all comments/suggestions.

1. "There is no real biological insight in the manuscript. The Authors do not really explain how MoDLE could be useful to understand the underlying biological processes. They advertise their tool as a fast alternative to existing tools in the field; they show how it can be used to make predictions but there is no strong message about inference. It is up to the Editor whether some biology is required. My opinion is that the article would be better if the Authors explained what question could be answered with MoDLE, and greatly better if they answered some of those questions."

We agree that providing more biological examples of uses of MoDLE is a good idea. We have now added two new use cases: i) inferring the occupancy of individual CTCF barriers [see point 2 below], and ii) predicting effects of mutations to CTCF barriers in the HoxD cluster. We have added paragraphs in the Results and Discussion sections where we explain the type of questions that can be answered with MoDLE. See Figures 5 and 6 and the new sections Predicting effects of TAD border alterations and Optimization of individual barrier parameters for details. We think that these changes have greatly improved our manuscript.

2. "It seems to me that the Authors could slightly modify their work in section "Genome wide parameter optimization" to somehow address the point above. If they let every CTCF site have their own value of P_BU (the dissociation rate), and fitted those values using the same Bayesian optimization procedure, they would obtain a CTCF binding profile. This is something they could compare to the actual profile and thereby verify that their model does allow to infer the underlying biology. This is not the best application because CTCF ChIP-seq is easy and Hi-C is hard, but at least this would show whether MoDLE can be used for inference in biology."

This is an excellent suggestion. We have now added a new biological example showing the optimization of each individual CTCF site in a region on chromosome 1. As the number of parameters to explore is quite large, we had to employ a different optimization strategy than for the genome-wide case (details are found in the Methods section). When we compare the resulting MoDLE output with the corresponding Micro- C data, as well as compare inferred binding profiles with actual ChIP-seq profiles, we indeed find a correspondence between these. See the new section Optimization of individual barrier parameters and Fig. 6 for details.

3. "The section "Genome wide parameter optimization" generated a lot of confusion in my mind."

We have now substantially revised this section to avoid confusion. See our point-by- point response to the reviewer’s specific comments below (numbering added by us).

4. "Line 243: "Notably, the results show that the near-optimal CTCF binding probabilities have values typically >0.8, except when the stability of binding (P) is high..."

What does that mean? The optimal value is 0.694, so what is typical about values greater than 0.8. Do the authors simply mean that when pi_B increases P_UU decreases? If so, why the complicated logic flow?"

We agree that this phrasing was confusing. We have now rephrased this part of the Results section. We meant to convey that a single, optimal (pi_B,P_UU) parameter combination displays very similar characteristics as other near-optimal parameter combinations. We find that both the CTCF occupancy (pi_B), as well as the binding stability (P_UU) can vary in a wide range between 0.6-0.8 and 0.1-0.95, respectively. In addition to changing the text, we have updated Fig. 4 (and added a Supplementary Fig. 11) showing more examples of near-optimal parameter combinations. We hope that these changes and additions will avoid confusion.

5. "Line 245: "... supporting a dynamic exchange model of CTCF binding and a transient maintenance of loops [42]".

How is that supporting this model exactly? The optimal value of P_UU 0.978 is close to the maximum possible so how is this dynamic? In other words, what kind of outcome would have been against a dynamic exchange model?"

We believe that with the revised text and updated Fig. 4C-D, we now clearly show that many parameter combinations can yield simulated heatmaps that are compatible with the observed micro-C data. The dynamic exchange model (as described in Hansen et al. 2017 [ref nr. 42]) posits a frequent CTCF exchange but with a low loop dissociation rate (i.e., stable loops). This is also what our range of near-optimal parameter combinations shows. If, for example, only a nontransient binding of CTCF (i.e. P_UU high) yielded optimal concordance with the observed data, this would oppose the dynamic exchange model. However, we believe that the word “supporting” in the text is slightly confusing, and have therefore changed this to: “compatible with”. We have also slightly rephrased this part of the text to hopefully improve its readability.

6. "Line 246: "Thus, CTCFs can potentially compensate for a lower CTCF binding probability by increasing "traffic jams" between extruding LEFs".

How is this a consequence of the previous statement? Also, I have a hard time understanding what the sentence means, starting from CTCF compensating for its binding probability, to the comparison lower with respect to what and finally the use of traffic jams between quotes, which seems to refer to a situation that is loosely defined."

We agree that the reference to the “traffic jam” hypothesis creates confusion, especially since the idea is somewhat loosely defined. We have therefore decided to remove this sentence from the manuscript.

7. "Figure 4A adds to the confusion. The two series of squares seem to represent the binding state of CTCF at barriers. Why are the bound sites more aggregated when P_UU + P_BB are high? I understand that the system becomes more static (fewer CTCF changes per epoch) in this case but according to the description of the model, CTCF binding is space-less (section 6 of supplementary document 1). So I do not understand how the spatial aggregation of occupancy comes about. I understand that when P_UU + P_BB are high, loop- extrusion factors spend more time at the barriers and may aggregate there, but this is not what the profiles are meant to represent (the legend says that it is the stationary distribution of pi_B)."

We see how Fig. 4A can be misinterpreted in its current form. Therefore, we have now labeled the figure to clarify what each box represents, and also expanded the figure legend accordingly. In brief, boxes do not illustrate a sequence of genomic bins, but rather the binding state of one CTCF binding site in a given simulation instance as the simulation progresses. Thus, the clustering shown in the figure is temporal, not spatial.

We hope the updated figure and figure legend make this clearer.

8. "Figure 2E: The authors should describe what "Accuracy" is."

We have now added an explanation in the figure legend, and in the main text . We also refer to the Methods for an in-depth explanation of how contact map pixels are classified and compared.

9. "Figure 4B: The authors should say something about the y axis. I understand that this is the standard output of scilearn-kit and the score function is fairly complex, but the plot is meaningless without this information. At the very least, the authors should link to the section describing the objective function."

We have now added a better description on the y-axis of Fig. 4B (it now says “Stripe dissimilarity score (lower is better)”. We have also added a description in the figure legend with reference to the Methods (part 6) section, where the comparison method is described in detail.

10. "The Conan troubleshooting info should be much more visible."

See below (point 11).

11. "The dependence on h5diff to run the test is not explicit in the manual."

We very much appreciate you reaching out on GitHub to raise these issues and hope you are happy with the way we have addressed them.

Both issues concern MoDLE’s documentation, not the software itself. In the first issue, reviewer #1 points out that the documentation could be improved by making troubleshooting information more visible e.g. by moving it closer to where issues are more likely to occur. We agree with reviewer #1 and have implemented their suggestions with pull request #39.

In the second issue, reviewer #1 reports that the h5diff tool, one of the dependencies of MoDLE’s automated test suite, is not mentioned in the documentation. After installing h5diff, reviewer #1 ran into another error, which was caused by the test suite assuming a recent version of h5diff is installed on the machine where the tests are being run. Pull request #38 updates the documentation as well as the test suite, which can now run even when using older versions of h5diff.

Reviewer #2

We thank the reviewer for the great suggestions for improving the manuscript. See below for our point-by-point response to all comments/suggestions.

1. "It would be interesting to present an application to evaluate the importance of different molecular mechanisms in relevant genomic loci. Specifically, it would be worth to consider other aspects known to be fundamental to chromatin architecture: what about phase-separation? Does the lack of this mechanism affect the accuracy of the prediction? Is the tool able to tell something about the role of each mechanism (LE and phase-separation) in some specific, biologically relevant examples?"

We agree that presenting applications of MoDLE to evaluate different molecular mechanisms is interesting. See also the comments from Reviewer #1. We have now added two example applications of MoDLE: i) inferring the occupancy of individual CTCF barriers, and ii) predicting effects of mutations to CTCF barriers in the HoxD cluster.

The second example showcases how MoDLE can be used to learn about barrier occupancies from the Hi-C data. We have also shown that MoDLE can be used to simulate CTCF/barrier occupancy (Fig. 6D) and Cohesin/LEF profiles (Fig. 6E) and compared these to corresponding ChIP-seq data.

The lack of e.g. phase separation and other forces driving genome compartmentalization will affect MoDLE’s output to a certain extent. This is mainly seen for contacts >2Mb in size, as shown in Supplementary Fig. 1 and the new Supplementary Fig. 3, where MoDLE is less accurate in capturing these interactions.

We have added a paragraph (see section Comparison with Micro-C data and MD simulations) where we describe the consequences of this effect in more detail.

2. "How the MoDLE predictions are related to other chromatin features, as A/B compartments?"

We have now computed and compared the MoDLE and OpenMM performance in A vs. B compartments (see Supplementary Fig. 5). As shown, MoDLE (similarly to OpenMM) displays minimal difference in accuracy in the two compartments. We reference this new comparison in the Results section.

3. "What about gene-enhancer contacts? Are there loci where the model can tell something about specific regulatory elements?"

This is indeed an interesting question. To show how MoDLE can be used to answer such (and related) questions, we have now included an example where we focus into a smaller region (the HoxD locus [GRCm38 chr2:74-75.5Mbp]) and model changes in looping upon partial and complete deletions of the barrier between the two domains (see Fig. 5). We can confirm that the full deletion (Fig. 5E) indeed causes a merge of the T- and C- domains and a rewiring of enhancer elements in this region.

We have added a more general paragraph in the Discussion where we discuss the possibility of modeling enhancer-promoter contacts.

4. "Does the model correctly recapitulates contact probability decay? Is there a critical genomic distance above which predictions are less reliable?"

We have added Supplementary Fig. 4 and an accompanying paragraph in the Results section exploring to which degree MoDLE recapitulates the observed contact probability decay. The default behavior of MoDLE does not recapitulate this trend fully. The reason is that MoDLE only samples loop extrusion contacts, and not e.g. inter- compartment contacts or random polymer contacts. However, we show that when the processivity is increased, this trend is closer to what is observed in Hi-C and Micro-C data (see Supplementary Fig. 4).

5. "I would evaluate the accuracy of the algorithm with other tests, e.g. if mutants phenotypes at a particular genomic distance can be correctly predicted."

In Fig. 2F we show modeled consequences of deleting barriers (CTCF) and reducing the chance that LEFs/Cohesin is unloaded from DNA (mimicking Wapl depletion). These results show expected consequences, as described in the results section. We have added a new example involving mutations in the HoxD locus (see comment 3 above, for details).

6. "Technical observations are about the evaluation of the accuracy. I think that other metrics should be used (distance corrected correlations, HiCRep from Yang et al 2017, correlations curve diagonal by diagonal) to estimate the similarity between MoDLE and experiments."

Very good suggestion. We have now added Supplementary Fig. 3, where we show correlations diagonal-by-diagonal in our selected regions, comparing with Micro-C data, but also with OpenMM (see Supplementary Fig. 3 for details).

7. "Also, more investigations about MoDLE output in varying the main LE parameters, i.e. LE density and processivity: how is the change of the pattern?"

Again, a very good point. We have now added Supplementary Fig. 7-10 investigating and showing consequences of varying loop extrusion parameters such as LE density and processivity. On a similar note, we have added more details and examples in Fig. 5, to show how the MoDLE output varies for different combinations of barrier/CTCF binding properties.

8. "Is the tool able to find the best LE parameter arrangement for a specific locus? Does the model result match with biological estimates of such quantities about Cohesin density and LEf processivity?"

These are relevant questions. We have now added an example where we optimize LE parameters for a specific locus on chromosome 1 (see Fig. 6), and compare with Rad21/cohesin and CTCF ChIP-seq data. See our comments to reviewer #1 point 2 for more details.

9. "Supp. 1 seems that long range is not well captured? Please comment on that."

It is true that long range contacts (~2-3Mbp) seen in Hi-C or Micro-C are not that well captured by MoDLE. We have added Supplementary Fig. 3 exploring this in more detail. The reason is that this range is dominated by compartmental and random polymer interactions, which MoDLE is not designed to model. MoDLE focuses on loop extrusion contact simulation. We have added a paragraph in the Results section explaining this in more detail.

10. "Beyond MicroC, a comparison with other data, such as HiC, would be useful?"

Great suggestion. We have now added comparisons with Hi-C data in Fig. 2D, 4D and Suppl. Fig 1 and 4. As can be seen from the plots, Micro-C mainly displays more resolved contact maps compared to Hi-C, allowing an easier comparison of the loop extrusion patterns.

11. "Add some synthetic and specific info in the page 7. How is the combination TAD contact - LE done? Please add some more details here with reference to the Methods."

We have now updated and improved the description of how this is done. See Section

9, Additional file 1 for details. We have also added a reference to this section in the Comparison with Micro-C data and MD simulations section.

Reviewer #3

We thank the reviewer for the suggestions for improving the manuscript by adding more biological context. See below for our point-by-point response to all comments/suggestions.

1. "The paper is particularly focus on the performance of this model and on thecnical details to prove that MoDLE is a more suitable choise compared to OpenMM. Only in the session "Comparison with Micro-C data and MD simulations" (and fig.2), the authors present the results of their model on five different 10 Mbp regions on five different chromosomes where the comparison with biological data seems limited to the median pixel accuracy."

We have now substantially revised the manuscript to include new biological examples showing use cases of MoDLE: i) predicting effects of mutations to CTCF barriers in the HoxD cluster (see Fig. 5) and ii) inferring the occupancy of individual CTCF barriers (see Fig. 6) . We have also included an additional way to compare with the data (see Supplementary Fig. 3), and also include new Hi-C data for comparison purposes. In our view, these new additions improve the quality and biological significance of our manuscript.

2. "The part of the model involving loop extrusion and CTCF introduces parameters for self-transitions and probability of a given CTCF to be occupied. The model can infer these parameters from ChIP-seq data or evaluate them using a Bayesian optimization. These parameters are not well contestualized in the biological dataset."

We apologize for not providing enough context, and for presenting this in a confusing way (see also comments from Reviewer 1, points 3-7). We have now substantially revised the figure and the Results section to make our points clearer.

3. "It is not clear what are the real differences between MoDLE and OpenMM: the authors present a comparison limited to the computational performances. What are the differences in terms of the model? Are their model for loop extrusion compatible with the one in OpenMM? What about the dynamical properties?"

This is a valid and important point. We have now added a better explanation of the actual model differences between MoDLE and OpenMM in the Results section. See also the Introduction section for details about the OpenMM model.

4. "This paper is really thecnical, focus in the perfomance improvements with a brief comparison with the biological context, It is not approrpaite for genome biology and should be send to focus journal dealing with software comparison performance. It is not for a broad audience of biologists."

We have made an effort to improve the biological context in the manuscript. In addition to the new added biological examples described in point 1, we expanded the Discussion section to describe and discuss potential uses of MoDLE in biological contexts.

**2^nd^ round**

**Reviewer 1**

The authors have addressed my concerns. I appreciate that the authors took my criticism constructively to improve their manuscript. The scientific quality of the manuscript is greatly improved in my opinion.

The only detail I would like to mention is that in the citations 43, 44 of page 11, the word "HoxD" has (weirdly) been deleted from the title. Maybe there was an erroneous find/replace at some point, or an issue with the display of the citations?

Otherwise and related to that, I recommend the authors to add a few indications to match the claims and their sources. For instance, the statement "known to cause an increase in interactions between the two domains" has no citation. It is in the next sentence as the citation 43,44 so I suppose the authors do not want to spam readers with bibliographic information... but here it would be nice to know in which of the two citations the interested researcher should look for the information. It's a detail but maybe the Authors could give one last pass to their text to make the coupling between statements and references a little tighter.

**Reviewer 2**

The authors addressed all my points and the paper results improved.

**Authors’ response**

Our response to the remaining comments from Reviewer 1:

- We have checked that all statements have correct citations. And everything is correct.

- The missing "HoxD" text in two of our citations have now been corrected.

- We have moved citations [43] and [44] to indicate which of the references that

support the claims about the increase in interactions and the rewiring of the enhancers.
